# A disentangled transformer-based transfer learning framework to predict patient drug response from tumor single-cell transcriptomics

**DOI:** 10.1093/bioinformatics/btag269

**Published:** 2026-07-07

**Authors:** Xinliang Sun, Li Shen, Linconghua Wang, Xinyi Zhang, Zhangli Lu, Jing Tang, Min Li

**Affiliations:** School of Computer Science and Engineering, Central South University, Changsha, Hunan 410083, China; Research Program in Systems Oncology, Faculty of Medicine, University of Helsinki, Helsinki 00290, Finland; School of Automation, Central South University, Changsha, Hunan 410083, China; School of Computer Science and Engineering, Central South University, Changsha, Hunan 410083, China; School of Computer Science and Engineering, Central South University, Changsha, Hunan 410083, China; Research Program in Systems Oncology, Faculty of Medicine, University of Helsinki, Helsinki 00290, Finland; School of Computer Science and Engineering, Central South University, Changsha, Hunan 410083, China

## Abstract

**Motivation:**

Intratumoral cellular heterogeneity limits therapeutic efficacy in cancer patients. Although single-cell transcriptomics offers high-resolution profiling, translating these insights into clinical drug response prediction remains challenging. Recently, transfer learning approaches have attempted to predict patient drug response by leveraging pre-clinical data. However, these approaches operate at the bulk level, often masking the cellular heterogeneity essential for prediction.

**Results:**

In this study, we propose scTAPE, a disentangled transfer learning framework to predict patient drug response using tumor single-cell transcriptomics. scTAPE follows a pre-training and fine-tuning paradigm. During the pre-training stage, scTAPE uses a disentangled learning strategy to extract intrinsic pharmacological signals masked by confounding factors from the matched bulk and single-cell expression profiles. Subsequently, a supervised drug response model is trained on labeled cell-line data to fine-tune the aligned common embedding, thereby achieving cross-domain generalization to unseen datasets. Experimental results demonstrate that scTAPE successfully predicts drug response across cell-line datasets and two independent clinical cohorts, outperforming state-of-the-art single-cell-based predictors. Furthermore, by analyzing tumor cell subpopulations, scTAPE not only predicts patient drug response to both single and combination treatments but also identifies potential therapeutic agents targeting drug-resistant subpopulations.

**Availability and implementation:**

The implementation of scTAPE is available via https://github.com/xinliangSun/scTAPE.

## 1 Introduction

Predicting clinical drug response from tumor transcriptomics is pivotal for precision oncology (Tsimberidou *et al.* 2022). Recent approaches have integrated large-scale pharmacological profiles to guide treatment (Ma *et al.* 2021, He *et al.* 2022, Zhou *et al.* 2025), but most still rely on bulk tumor data. This reliance obscures intratumoral heterogeneity, which drives clonal selection, drug resistance, and tumor recurrence (Bhat *et al.* 2024). Although single-cell RNA sequencing (scRNA-seq) offers high-resolution insights into this molecular heterogeneity, translating scRNA-seq data into effective therapeutic predictions remains a significant challenge.

Several computational approaches have emerged to predict single-cell drug sensitivity by exploiting bulk pharmacogenomic data. For example, DREEP (Gambardella *et al.* 2022) and scDR (Lei *et al.* 2023) directly map cell-line derived signatures onto scRNA-seq data. Precily (Chawla *et al.* 2022) and scPDS (Yao *et al.* 2025) utilize shared pathway features to bridge the gap between bulk and single-cell modalities. However, these approaches often fail to account for the inherent distributional shift between bulk and single-cell transcriptomes. Deep transfer learning models like scDEAL (Chen *et al.* 2022) and SCAD (Zheng *et al.* 2023) have been developed to align distributions using maximum mean discrepancy (MMD) or adversarial learning. However, these approaches require access to the target domain during training, and their application has so far been restricted to preclinical models with limited validation in real-world clinical contexts.

Recent advancements have focused on tailoring patient treatment by harnessing single-cell tumor transcriptomics. For example, Beyondcell (Fustero-Torre *et al.* 2021) utilizes drug-induced expression signatures to identify tumor subclones with distinct drug sensitivities, proposing cancer-specific treatments from scRNA-seq data. scTherapy (Ianevski *et al.* 2024) leverages pharmacological perturbation profiles to train a machine learning model that prioritizes therapeutic compounds capable of selectively targeting specific cellular subpopulations within individual patients. To address generalization challenges, PERCEPTION (Sinha *et al.* 2024) incorporates transfer learning principles, utilizing paired single-cell data to fine-tune bulk-trained models for clone-level prediction.

Despite methodological progress, the effective transfer of drug response patterns is still hindered by the substantial discrepancy between bulk and single-cell transcriptomes. A major challenge is that the robust pharmacogenomic signatures derived from bulk data are often confounded by the intrinsic noise and heterogeneity of single-cell profiles. Consequently, conventional global alignment strategies (e.g., MMD or adversarial learning approaches) fail to decouple true therapeutic effects from modality-specific variance, thereby constraining the model’s generalization capability at single-cell resolution.

To address this challenge, we present scTAPE, a disentangled transformer-based framework tailored for predicting patient drug response from tumor single-cell transcriptomics. scTAPE adopts a two-stage pre-training and fine-tuning paradigm. During pre-training, scTAPE uses a domain separation strategy to disentangle confounding factors from shared pharmacological signals, while leveraging a gradient-penalized Wasserstein Generative Adversarial Network (WGAN) (Gulrajani *et al.* 2017) to enforce local alignment of intrinsic drug response signals. Subsequently, a supervised predictor is trained on labeled cell-line data to refine the aligned embeddings, enabling generalization to unseen target domains.

To bridge the gap between single-cell data and clinical outcomes, we also introduce a heuristic clone-aware patient scoring strategy for high-resolution patient-level prediction. Experimental results demonstrate that scTAPE successfully predicts drug response on cell-line data and independent clinical cohorts, outperforming state-of-the-art single-cell-based predictors. Furthermore, by dissecting tumor cell subpopulations, scTAPE not only predicts patient response to monotherapies and combinatorial regimens but also identifies potential therapeutic agents targeting drug-resistant clones. The main contributions of this work are summarized as follows:

- We propose a disentangled transformer-based transfer learning framework to extract intrinsic pharmacological signals masked by confounding factors.
- Our two-stage pre-training and fine-tuning strategy facilitates robust generalization to unseen target domains.
- Experimental results on cell-line datasets and two independent clinical cohorts demonstrate that scTAPE outperforms state-of-the-art single-cell predictors.

## 2 Materials and methods

**Figure 1:**
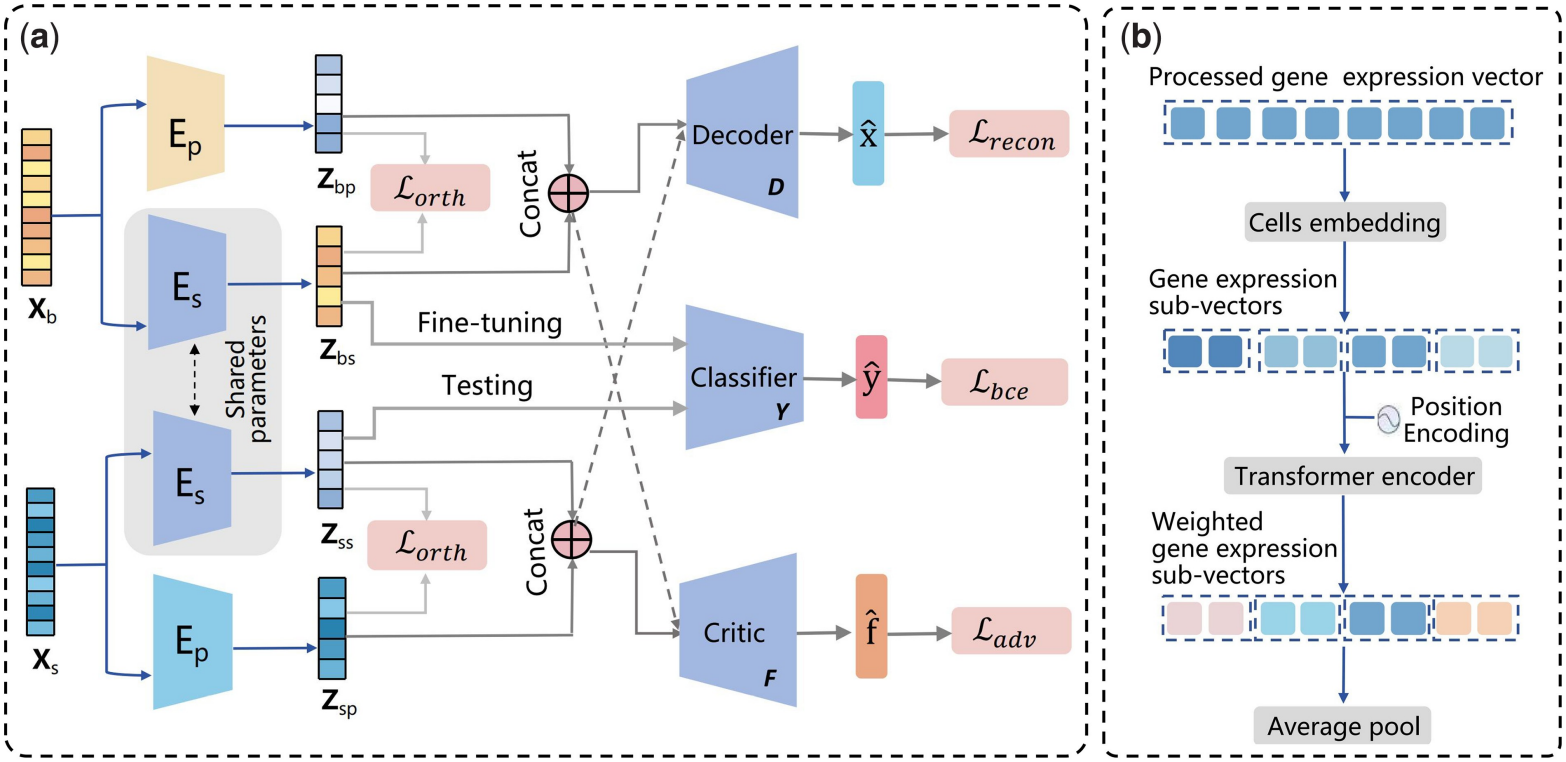
Overview of the scTAPE framework. (a) scTAPE operates under a two-stage paradigm. First, during pre-training, it utilizes a transformer-based encoder to extract high-level gene expression features. Second, during fine-tuning, a supervised predictor is trained using drug-specific labeled cell-line data to optimize the aligned embeddings for downstream tasks. (b) Detailed diagram of the encoder module.

In this section, we first describe the benchmark datasets used in the proposed model. We then introduce the scTAPE model framework. As illustrated in Fig. 1a, the framework is built upon a transformer-based encoding component *E*, which consists of a shared encoder $E_{s}$ and domain-specifice private encoders collectively denoted as $E_{p}$. Specifically, $E_{s}$ extracts common representations from both bulk and single-cell data, while $E_{p}$ captures domain-specific attributes from each corresponding domain. The architecture also includes a decoder *D*, composed of a two-layer multilayer perceptron (MLP), which maps latent embeddings back to the original gene expression space. To ensure domain alignment, a critic *F* uses adversarial learning to regularize the feature distributions of bulk and single-cell data. Finally, a classifier *Y* leverages these shared, regularized features to make accurate predictions. This design effectively facilitates the transfer of knowledge from the bulk cell-line domain to the single-cell level. Figure 1b depicts the detailed diagram of the encoder *E*.

### 2.1 Data preparation

We retrieved RMA-normalized pan-cancer bulk RNA-seq profiles from the CCLE (Barretina *et al.* 2012) and the corresponding area under the dose-response curve (AUC) values from the PRISM project (Corsello *et al.* 2020). Matched single-cell UMI count matrices for cell lines were obtained from the Broad Single-Cell Portal (Kinker *et al.* 2020), while independent cell line and clinical scRNA-seq cohorts for validation were curated from DRMref (Liu *et al.* 2024) and GEO. The data summary is briefly shown in Table 1. The overlapping cell lines between the single-cell datasets and the CCLE data used in model training and evaluation are summarized in Table 1, available as supplementary data at *Bioinformatics* online. To standardize drug-response labels across drugs, we z-score normalized the AUC values for each drug across all cell lines and used *z* = 0 as a uniform binarization cutoff. Cell lines with *z*-scored AUC < 0 were labeled as sensitive 1, whereas those with z-scored AUC > 0 were labeled as resistant 0. We retained the original preprocessing for datasets with existing quality control (QC). Raw datasets without prior QC were processed using a standardized Scanpy pipeline (Wolf *et al.* 2018). For model input, we independently selected the top 5000 highly variable genes from the bulk and single-cell data and took their union, yielding a unified feature space of 6281 genes. For clinical cohorts data, we included human cancer scRNA-seq datasets with treatment response annotations and at least 10 evaluable samples.

**Table 1:** Summary of the scRNA-seq datasets.

Drug	Source	GEO accession	Cancer type
Dasatinib	Cell line	GSE158457	Acute lymphoblastic Leukemia
Docetaxel	Cell line	GSE140440	Prostate cancer
Erlotinib	Cell line	GSE149214	Lung cancer
PLX-4720	Cell line	GSE108383	Melanoma
Paclitaxel	Patient	GSE169246	Breast cancer
Bortezomib Melphalan Prednisolone	Patient	GSE189460	Multiple myeloma

### 2.2 Transformer-based encoder for gene expression

To capture coordinated gene expression programs while improving computational efficiency, we use a cell embedding strategy adapted from (Xu *et al.* 2023) to construct the model input. Formally, let a gene expression profile be denoted as a vector $x\in R^{v}$, comprising *v* highly variable genes. We first segment x into a sequence of *M* sub-vectors (tokens) of length *k*, denoted as $T=\{t_{0},\ldots ,t_{M-1}\}$, where the *j*-th token corresponds to $t_{j}=[x_{j\cdot k},\ldots ,x_{j\cdot k+k-1}]$ and M=⌈v/k⌉. Notably, before sub-vector partitioning, genes are aligned according to a fixed lexicographic order of gene symbols. Sinusoidal positional encodings (*PE*) are then added element-wise to the tokens, yielding the input sequence $H^{\left(0\right)}=T+PE$. The encoding for a position *pos* and dimension index *d* is defined as:


(1) $$PE(\mathrm{pos},d)=\{\begin{array}{cc} \;\text{sin}(\mathrm{pos}/10000^{d/k}) & \text{if}\;d\;\text{is}\;\text{even}\\ \;\text{cos}(\mathrm{pos}/10000^{(d-1)/k}) & \text{if}\;d\;\text{is}\;\text{odd} \end{array}$$

The encoder E(·) consists of stacked Transformer layers. Central to this architecture is the Multi-Head Self-Attention (MSA) mechanism. For an intermediate representation H, the attention function computes the weighted relevance of gene tokens:


(2) $$\text{Attention}(Q,K,V)=\text{softmax}\left(\frac{QK^{T}}{\sqrt{d_{k}}}\right)V$$

where *Q, K, V* are linear projections of H. The processing within the *l*-th Transformer layer includes MSA and a Feed-Forward Network (FFN), integrated with residual connections and layer normalization (LN):


(3) $$\begin{array}{c} H^{\prime }=\text{LN}\left(H^{\left(l-1\right)}+\text{MSA}(H^{\left(l-1\right)})\right)\\ H^{\left(l\right)}=\text{LN}\left(H^{\prime }+\text{FFN}(H^{\prime })\right) \end{array}$$

Finally, to obtain a unified cell embedding $z\in R^{d}$, we apply global average pooling across all tokens of the final layer output:


(4) $$z=E(x)=\frac{1}{M}\sum_{j=0}^{M-1}h_{j}^{\left(L\right)}$$

This formulation E(·) serves as the backbone architecture for the domain-specific encoders described in the subsequent section.

### 2.3 Representation disentanglement via dual encoders

To effectively integrate bulk and single-cell data, we propose a disentanglement framework that separates domain-invariant biological signals from domain-specific variations. Let $X_{b}\in R^{N_{b}\times v}$ and $X_{s}\in R^{N_{s}\times v}$ represent the bulk and single-cell gene expression matrices, respectively, consisting of $N_{b}$ and $N_{s}$ samples with *v* genes. We feed these inputs into two distinct encoders: a weight-sharing encoder $E_{s}$ and domain-specific private encoders collectively denoted as $E_{p}$. The shared encoder $E_{s}$ is designed to extract common pharmacological signals, while the private encoder $E_{p}$ captures domain-specific attributes from their corresponding domains.

Specifically, taking the bulk dataset $X_{b}$ as input yields two embeddings: $Z_{bs}=E_{s}(X_{b})$ and $Z_{bp}=E_{p}(X_{b})$. Similarly, the single-cell dataset $X_{s}$ is encoded as $Z_{ss}=E_{s}(X_{s})$ and $Z_{sp}=E_{p}(X_{s})$. To ensure that the generated shared embedding and the private embeddings do not capture redundant information, we introduce an orthogonal loss $L_{\mathrm{orth}}$ between them:


(5) $$L_{\mathrm{orth}}={\left|\left|Z_{bs}^{T}Z_{bp}\right|\right|}_{F}^{2}+{\left|\left|Z_{ss}^{T}Z_{sp}\right|\right|}_{F}^{2}$$

For model training, we concatenate the shared and private embeddings before passing them through a shared decoder *D*, composed of MLP. The reconstruction process is formulated as:


(6) $${\hat{X}}_{b}=D(Z_{bs}⊕Z_{bp}),\;{\hat{X}}_{s}=D(Z_{ss}⊕Z_{sp})$$

where ${\hat{X}}_{b}$ and ${\hat{X}}_{s}$ are the reconstructed profiles, and ⊕ denotes vector concatenation. We measure the reconstruction quality using the mean squared error:


(7) $$L_{\mathrm{recon}}=\frac{1}{N_{b}}\sum_{i=1}^{N_{b}}{\left|\left|x_{b}^{\left(i\right)}-{\hat{x}}_{b}^{\left(i\right)}\right|\right|}^{2}+\frac{1}{N_{s}}\sum_{i=1}^{N_{s}}{\left|\left|x_{s}^{\left(i\right)}-{\hat{x}}_{s}^{\left(i\right)}\right|\right|}^{2}$$

### 2.4 Domain adaptation

To achieve efficient generalization of the downstream model, the generated representation needs to be invariant across different domains. Inspired by the previous works (He *et al.* 2022, Zhai and Liu 2024), we utilize the WGAN to train a critic network *F*. The role of this critic network is to distinguish between representations from two different domains. Meanwhile, an encoder attempts to generate embeddings that can fool the critic. In this way, the critic network and encoder will play a min-max game following an alternating training schedule. Specifically, the critic network *F* takes as input the concatenation of shared and private representation from different data sources. $L_{\mathrm{critic}}$ aims to maximize the difference between the mean critic scores for bulk pan-cancer data $F(Z_{b})$ and single-cell pan-cancer data $F(Z_{s})$. Conversely, $L_{\mathrm{gen}}$ consistently gives higher critic scores for representations of single-cell pan-cancer data. To avoid unstable training procedure, we introduce a gradient penalty term (Gulrajani *et al.* 2017) to encourage the gradient of the critic to have a norm close to 1. Mathematically, the aforementioned adversarial loss is defined as follows:


(8) $$L_{\mathrm{adv}}:\{\begin{array}{cc} L_{\mathrm{critic}}=\frac{1}{N_{s}}\sum_{i=1}^{N_{s}}F(Z_{s}^{i})-\frac{1}{N_{b}}\sum_{i=1}^{N_{b}}F(Z_{b}^{i}) & \\ \;\;\;+\lambda {\left(||\nabla_{\bar{Z}}F(\bar{Z})|{|}_{2}-1\right)}^{2} & \\ L_{\mathrm{gen}}=-\frac{1}{N_{s}}\sum_{i=1}^{N_{s}}F(Z_{s}^{i}) & \end{array}$$

where $Z_{s}$ and $Z_{b}$ denote $Z_{ss}⊕Z_{sp}$ and $Z_{bs}⊕Z_{bp}$, respectively. $\bar{Z}=\varepsilon Z_{b}+(1-\varepsilon )Z_{s}$, and ε∼U(0,1), a standard uniform distribution exclusive between 0 and 1.

### 2.5 Model training

The training procedure of our framework is structured into two sequential phases: unsupervised pre-training and supervised fine-tuning. In the pre-training phase, we leverage transcriptomic profiles from both bulk and single-cell pan-cancer datasets to optimize the autoencoder, domain separation network, and domain adaptation module. To ensure training stability and robust feature alignment, we adopt a stepwise optimization strategy. Initially, the model is trained solely with reconstruction and orthogonality constraints ($L_{\mathrm{recon}}+L_{\mathrm{orth}}$) to establish stable feature representations. Subsequently, the adversarial loss is integrated to enforce domain invariance, resulting in a comprehensive pre-training objective defined as


(9) $$L_{\mathrm{pre}}=L_{\mathrm{adv}}+L_{\mathrm{recon}}+L_{\mathrm{orth}}$$

Subsequently, in the fine-tuning phase, the pre-trained shared encoder is augmented with a task-specific MLP for drug response prediction. The entire network is fine-tuned on labeled bulk pan-cancer samples by minimizing the binary cross-entropy loss ($L_{\mathrm{bce}}$). To mitigate overfitting during fine-tuning, the dataset is stratified into training (80%) and validation (20%) sets, with early stopping performed according to validation metrics. During testing, the optimized model is applied to single-cell data, thereby enabling the transfer of therapeutic knowledge from bulk tissues to cellular resolution. Notably, the model is drug-specific and must therefore be retrained for each drug separately.

### 2.6 Heuristic clone-aware patient scoring strategy

To bridge the gap between single-cell data and clinical outcomes, we also introduced a heuristic clone-aware patient scoring strategy for high-resolution patient-level prediction. Specifically, for each patient *p*, malignant cells were partitioned into transcriptional clones $C_{p}$ using Louvain clustering (Blondel *et al.* 2008) (resolution = 0.8). We then characterized each clone $c\in C_{p}$ by computing a pseudo-bulk centroid (mean expression profile), which served as the input for scTAPE to predict drug response.

For a given drug *d*, scTAPE generates a predicted sensitivity score for each clone, denoted as ${\hat{y}}_{c,d}$. We define the raw patient-level response $r_{p,d}$ based on the sensitivity of the most resistant clone (i.e. the lowest predicted sensitivity among all clones):


(10) $$r_{p,d}=\underset{c\in C_{p}}{\min}{\hat{y}}_{c,d}.$$

To enable cross-drug comparability, we standardize $r_{p,d}$ across the cohort using z-score transformation:


(11) $$Z_{p,d}=\frac{r_{p,d}-\mu_{d}}{\sigma_{d}},$$

where $\mu_{d}$ and $\sigma_{d}$ represent the cohort mean and standard deviation of $r_{p,d}$, respectively.

For combination therapies involving a drug set D, we aggregate the standardized responses using a minimax strategy, consistent with the Independent Drug Action (IDA) principle (Plana *et al.* 2022):


(12) $$R_{p}^{\left(D\right)}=\underset{d\in D}{\max}Z_{p,d}.$$

Intuitively, this formulation identifies the agent most effective at overcoming the patient’s intrinsic resistance relative to the population, while maintaining the conservative assumption that the most resistant clone constrains the overall therapeutic efficacy.

## 3 Results

### 3.1 Baseline methods

To evaluate the performance of our proposed model, we compared scTAPE with state-of-the-art methods for drug response prediction at both the single-cell and patient levels. For methods with available hyperparameter ranges, we performed model tuning accordingly, whereas for methods without such information, we adopted the default settings reported in the original papers.

- By projecting bulk and single-cell gene expression data into shared pathway features, Precily (Chawla *et al*. 2022) can robustly predict drug response at single-cell resolution.
- scDEAL (Chen *et al*. 2022) utilizes the MMD approach to align the distributions of bulk and single-cell data to achieve drug response prediction at the single-cell level.
- SCAD (Zheng *et al*. 2023) utilizes adversarial learning strategy to infer the drug sensitivities at single-cell level.
- Beyondcell (Fustero-Torre *et al*. 2021) identifies tumor subclones with distinct drug responses from scRNA-seq data, using drug-induced expression signatures to propose cancer-specific treatments.
- PERCEPTION (Sinha *et al*. 2024) uses paired single-cell data to optimize hyperparameters in a bulk-trained machine learning model to predict drug response at the clone level.

### 3.2 scTAPE accurately predicts single-cell drug responses

**Figure 2:**
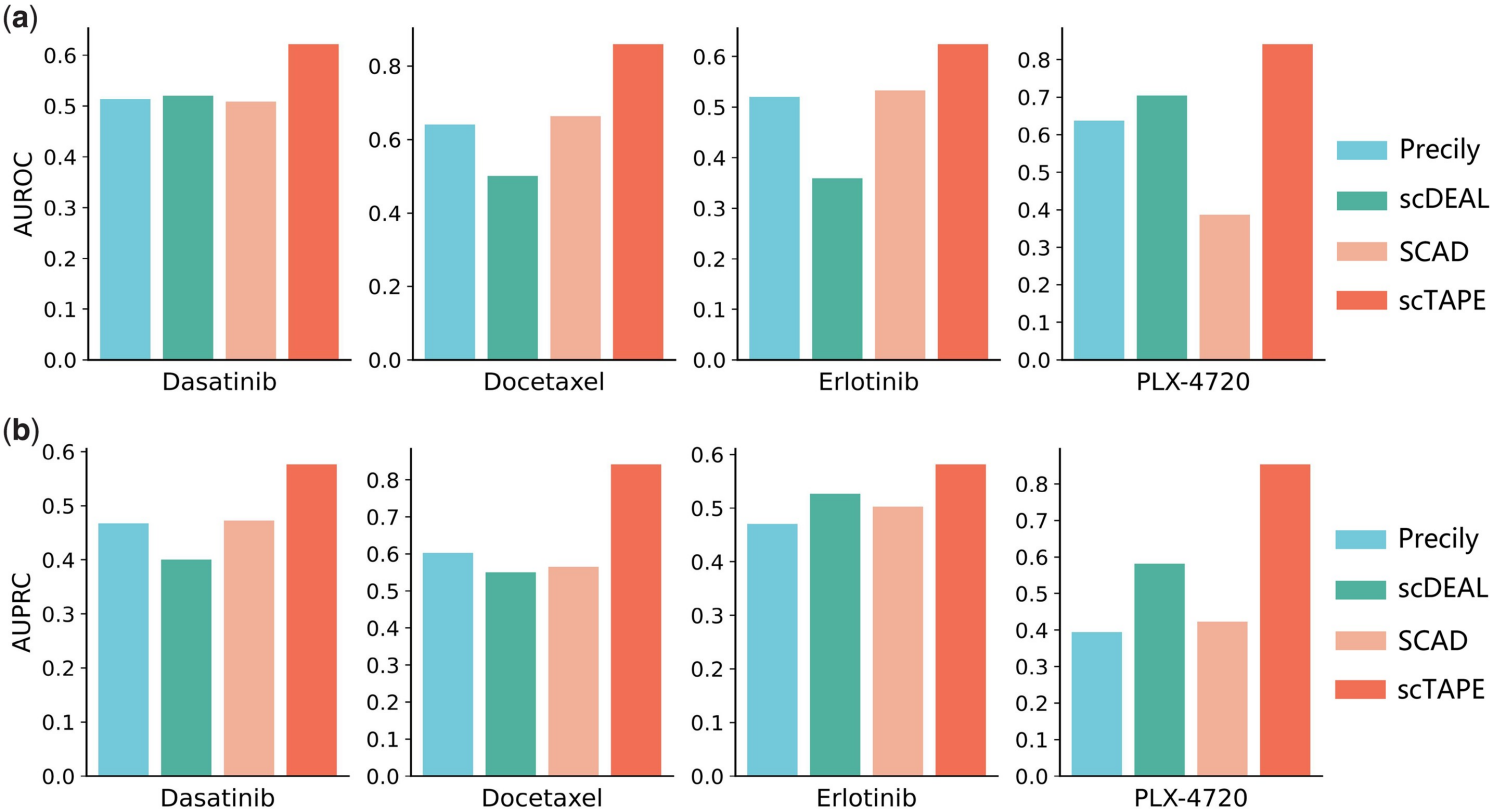
The performance of different methods in predicting single-cell drug response across four datasets as measured by AUROC (a) and AUPRC (b).

To comprehensively evaluate model performance, we first benchmarked scTAPE against three deep learning–based predictors (Precily, scDEAL, and SCAD) using four public scRNA-seq cell-line datasets generated under treatment with Dasatinib, Docetaxel, Erlotinib, and PLX-4720. We reported scTAPE and the baseline methods using the area under the receiver operating characteristic curve (AUROC) and the area under the precision–recall curve (AUPRC). As shown in Fig. 2, scTAPE consistently outperformed all competing methods across the four datasets on both metrics. In particular, scTAPE achieved the highest AUROC on the Dasatinib, Docetaxel, Erlotinib, and PLX-4720 datasets, exceeding the second-best method by 10.14%, 19.59%, 9.16%, and 13.63%, respectively. A similar trend was observed for AUPRC, where scTAPE improved upon the second-best method by 10.35%, 23.85%, 5.50%, and 27.17%, respectively. We additionally evaluated our model by adopting an scGPT-based model (Cui *et al.* 2024) as the pretrained backbone. Although the scGPT-based variant outperformed several baseline methods, it still failed to match the performance of the original scTAPE-based version (Fig. 1c and d, available as supplementary data at *Bioinformatics* online). These results suggest that, while a general single-cell foundation model is able to provide useful representations, it is still less effective than our original design. We also observed that the baseline methods generally yielded comparatively lower AUPRC values, which may be associated with data imbalance and domain shift and their potential effects on predictive accuracy.

**Figure 3:**
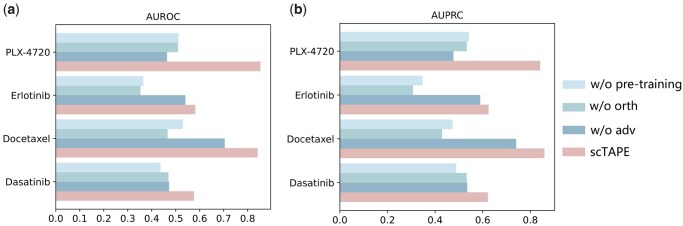
Comparison of scTAPE and its variant across four datasets in terms of AUROC (a) and AUPRC (b).

To further evaluate the contribution of each component within scTAPE, we performed ablation studies by removing pre-training, the orthogonal regularization term, and adversarial learning mechanism, respectively. As shown in Fig. 3, the integration of adversarial learning yielded consistent performance gains across all datasets, resulting in average improvements of 15.12% in AUROC and 16.8% in AUPRC. These results suggest that adversarial learning improves the generalization capabilities of the model. Furthermore, removing either the pre-training strategy or the orthogonality term resulted in a performance drop, underscoring their importance in mitigating biological noise and promoting the alignment of domain-invariant features. We also performed parameter sensitivity analyses on the number of HVGs and the subvector length, and found that the model achieved the best performance when the number of HVGs was set to 5000 and the subvector length was set to 512. The hyperparameter settings for scDEAL and SCAD are provided in Tables 2 and 3, available as supplementary data at *Bioinformatics* online.

### 3.3 Predicting drug response in a multiple myeloma clinical cohort

To assess the clinical applicability of scTAPE, we extended our evaluation to clinical outcome stratification using pre-treatment single-cell RNA-seq data from bone marrow aspirates of 18 multiple myeloma (MM) patients receiving bortezomib-based combination therapy (bortezomib, melphalan, and prednisolone). We first identified tumor transcriptional clones from each patient’s single-cell expression profiles and then predicted treatment response for each clone individually. Building on previous work (Sinha *et al.* 2024), we further developed and assessed five clone-level aggregation strategies to infer patient-level clinical response and identify the most effective approach.

**Figure 4:**
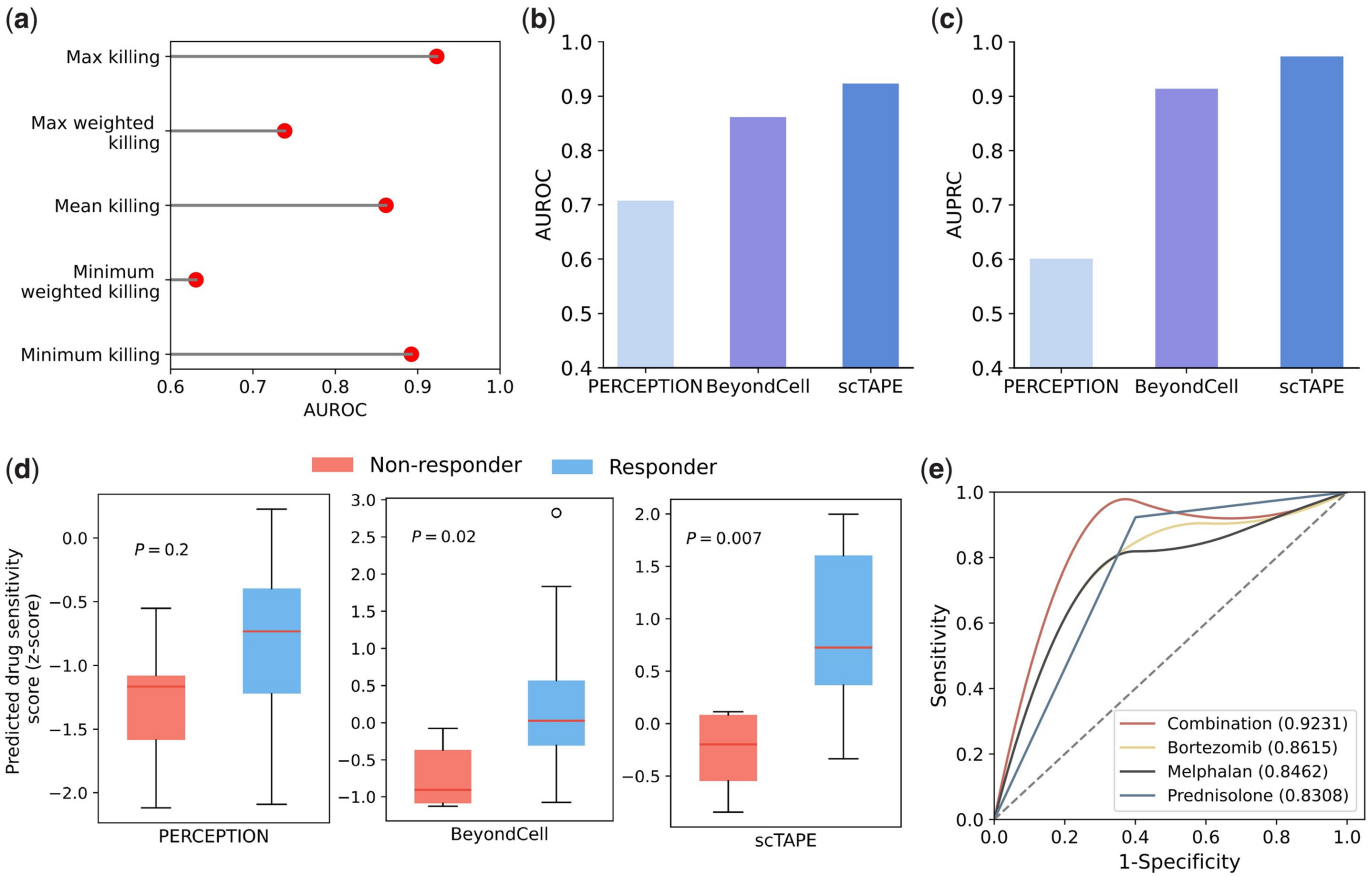
Prediction of scTAPE for bortezomib-based combination therapy in multiple myeloma patients. (a) The AUROC performance of five strategies in predicting patient drug response based on clone level predicted scores. (b) Performance comparison of different method in AUROC for predicting the clinical response in multiple myeloma patients. (c) Performance comparison of different method in AUPRC for predicting the clinical response in multiple myeloma patients. (d) The predicted combination response in 18 multiple myeloma patients stratified by non-responder versus responder status. A one-sided Wilcoxon rank-sum test was performed to compare groups. (e) The receiver operating characteristic curve showing the predicted combination response and monotherapy response.

We considered five strategies to aggregate clone-level predictions into a patient-level clinical response: (i) most-resistant clone, defined as the clone with the lowest predicted response score; (ii) abundance-weighted most-resistant clone, where the most-resistant clone’s score is weighted by its estimated abundance; (iii) mean clone response, computed as the average predicted score across all clones; (iv) most-sensitive clone, defined as the clone with the highest predicted score; and (v) abundance-weighted most-sensitive clone, where the most-sensitive clone’s score is weighted by its abundance in the tumor. Among these strategies, the most-resistant clone achieved the highest AUROC (0.9231), followed by the mean clone response (0.8615), abundance-weighted most-resistant clone (0.7385), most-sensitive clone (0.7077), and abundance-weighted most-sensitive clone (0.6308) (Fig. 4a). This pattern may reflect that therapy-resistant clones in multiple myeloma can be preferentially selected during treatment and become dominant, thereby better capturing patient-level clinical outcomes.

Using the most-resistant-clone scores, scTAPE accurately predicted treatment response in MM patients, achieving an AUROC of 0.9231 (Fig. 4b). This performance exceeded that of BeyondCell and PERCEPTION by 6.16% and 21.54%, respectively. scTAPE also achieved an AUPRC of 0.9746, outperforming the second-best method BeyondCell by 3.22% (Fig. 4c). Consistent with these results, scTAPE predicted higher sensitivity in responders than in non-responders (one-sided Wilcoxon rank-sum test, *P *=0.003; Fig. 4d), supporting the validity of the proposed clinical response scoring strategy in the MM cohort. We further trained drug-specific models for each agent in the combination regimen (Fig. 4e). Notably, interpolation was used only for visualization at the plotting stage to improve figure clarity, and no smoothing was applied to the underlying ROC data. Overall, single-agent predictions were less accurate than the combination-response prediction. Among the individual agents, the proteasome inhibitor bortezomib showed the best performance (AUROC = 0.8615). These findings are consistent with the clinical benefit of combination therapy and underscore the central role of bortezomib in first-line MM treatment. Based on these results, we used the most-resistant-clone strategy as the default for predicting patient-level clinical response in subsequent analyses. We further conducted a parameter sensitivity analysis on the resolution and observed that the model performed best when the resolution was set to 0.8 (Fig. 2a, available as supplementary data at *Bioinformatics* online).

### 3.4 Predicting chemotherapy response in a breast cancer cohort

Applying our most-resistant clone response strategy, we further evaluated scTAPE’s predictive generalization in a Triple-Negative Breast Cancer (TNBC) clinical trial cohort (Zhang *et al.* 2021). This study enrolled 22 patients with advanced TNBC, stratified into two treatment arms: paclitaxel monotherapy and combination therapy (atezolizumab plus paclitaxel). For the TNBC cohort, we selected nine patients in the chemotherapy arm with available pre-treatment scRNA-seq data and corresponding post-treatment clinical response annotations. To increase the sample size and improve the robustness of the model predictions, we additionally included distinct tumor samples derived from diverse tissues within these patients, yielding a final dataset of 15 tumor samples.

**Figure 5:**
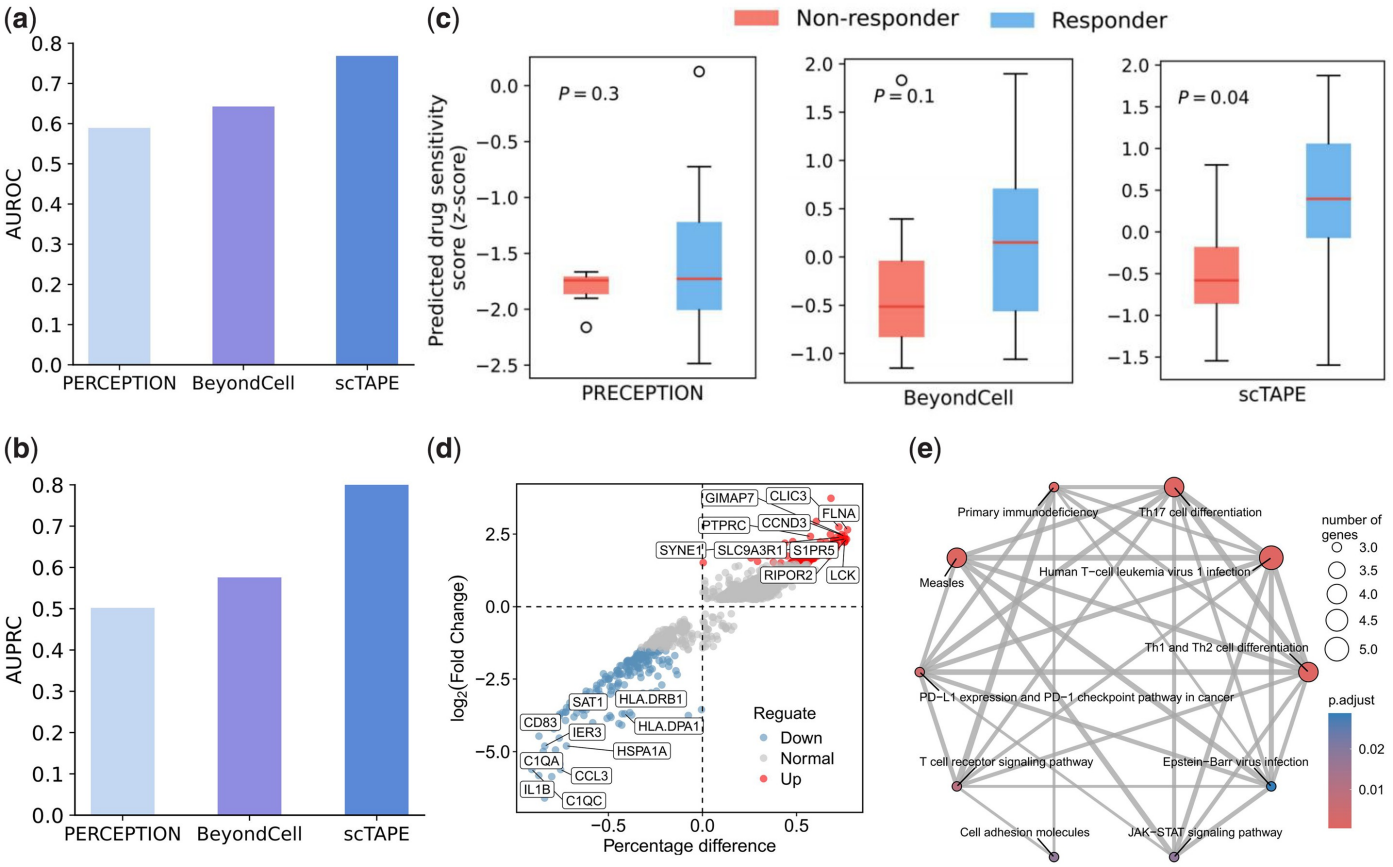
scTAPE prediction of chemotherapy response in the TNBC clinical trial. (a) Performance comparison of TNBC clinical response classification based on AUROC. (b) Performance comparison of TNBC clinical response classification based on AUPRC. (c) The predicted chemotherapy response in TNBC patients stratified by non-responder versus responder status. A one-sided Wilcoxon rank-sum test was performed to compare groups. (d) Differential expression gene (DEG) between responders and non-responders based on the most resistant clone as predicted by scTAPE. Genes with significantly higher expression in non-responders are positioned in the upper right, while genes with significantly lower expression in responders are positioned in the lower left. The top 10 significant genes are labeled. (e) KEGG enrichment analysis of the top upregulated genes in non-responders.

We built a clinical model for predicting patient response based on pre-treatment data from TNBC patients. The comparison of model performance is shown in Fig. 5a. scTAPE achieved the highest AUROC of 0.75, demonstrating superior performance in predicting patient response. In contrast, BeyondCell and PERCEPTION exhibited lower predictive accuracy with AUROC values of 0.6786 and 0.5714, respectively. In terms of AUPRC, scTAPE achieved a score of 0.8325, which was 12.89% higher than that of the suboptimal model BeyondCell (Fig. 5b). Compared to baseline methods, the predicted sensitivity scores of non-responders by scTAPE were significantly lower than those of responders (Wilcoxon rank-sum test, one-sided *P *=0.04; Fig. 5c). We further conducted a parameter sensitivity analysis on the resolution and observed that the model performed best when the resolution was set to 0.8 (Fig 2b, available as supplementary data at *Bioinformatics* online).

**Figure 6:**
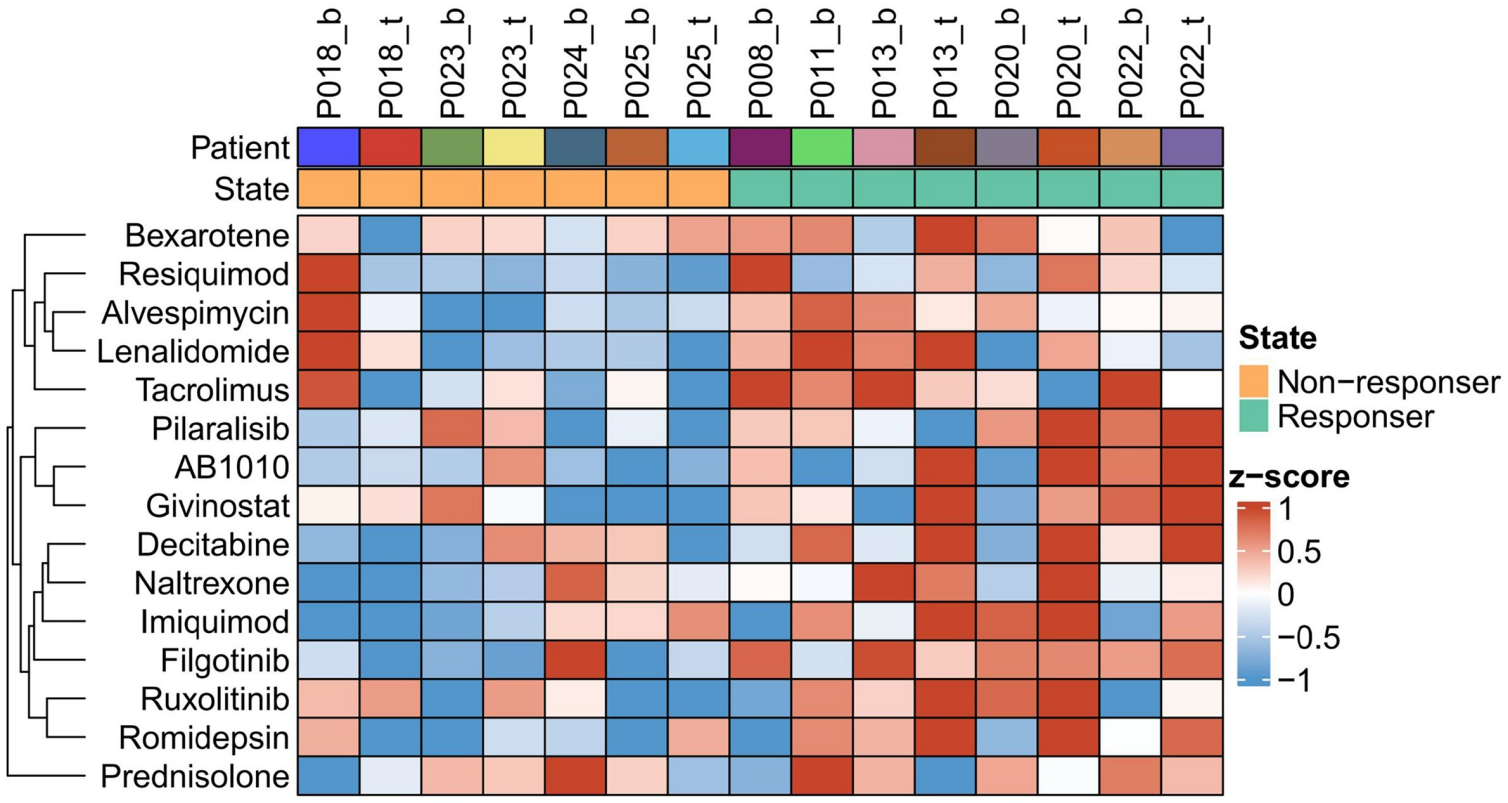
Heatmap of scTAPE-predicted scores for 15 immune-related drugs in the TNBC patient cohort. Labels above columns indicate sample source (_b: blood; _t: tumor).

To characterize transcriptional programs associated with heterogeneous treatment response, we conducted patient-level differential expression analysis between responders and non-responders based on the most resistant clones inferred by scTAPE (Fig. 5d). Among the top upregulated genes in the paclitaxel-resistant group, FLNA, SLC9A3R1, and CLIC3 were identified, each of which has been linked to chemotherapy resistance in TNBC (Macpherson *et al.* 2012, Qiang *et al.* 2014, Zhao *et al.* 2016). In addition, higher CLIC3 expression has been associated with poorer prognosis, supporting its potential relevance as a predictive biomarker (Macpherson *et al.* 2012). KEGG pathway enrichment analysis provided further context for these signatures (Fig. 5e), with enrichment patterns implicating immune-related processes, consistent with the original study (Zhang *et al.* 2021). Guided by these results, we next evaluated 15 immune-modulating agents with scTAPE. Overall, responders tended to show higher predicted sensitivity to these drugs (Fig. 6). Notably, bexarotene, prednisolone, ruxolitinib, and decitabine showed higher predicted sensitivity in non-responders, indicating potential options for paclitaxel-refractory patients. Consistent with this, bexarotene and ruxolitinib have advanced to Phase I/II evaluation in TNBC (Thomas *et al.* 2023, Lynce *et al.* 2024), and decitabine has been assessed in prospective clinical studies (Wang *et al.* 2024).

Details of the differential expression and enrichment analyses are provided below. Differential gene expression analysis was performed using Seurat’s FindMarkers() function with the Wilcoxon rank-sum test, followed by Bonferroni correction for multiple testing. Genes with $|{\;\text{log}\;}_{2}\text{FC}|\geq 1$ and an adjusted *P* value ≤ 0.05 were considered differentially expressed. KEGG enrichment analysis was conducted using clusterProfiler::enrichKEGG based on the top 100 significantly upregulated genes in non-responders, ranked by adjusted *P* value. Pathways with *P* ≤ 0.05 and q≤0.05 were considered significantly enriched, with multiple testing correction performed using the Benjamini–Hochberg method.

## 4 Conclusion

In this study, we introduced scTAPE, a disentangled transformer-based transfer learning framework designed to predict patient therapeutic responses at single-cell resolution. By leveraging a disentangled autoencoder architecture and a two-stage alignment strategy, scTAPE effectively bridges the domain gap, facilitating the transfer of pharmacological knowledge from bulk screenings to single-cell transcriptomes. Comprehensive benchmarking demonstrates that scTAPE not only achieves superior accuracy in single-cell response prediction but also outperforms state-of-the-art methods on independent clinical patient cohorts. Furthermore, ablation studies validated the architectural necessity of each model component. Besides, scTAPE exhibits clinical utility in optimizing both monotherapies and combinatorial regimens, thereby expanding the toolkit for precision oncology. Despite these advancements, our current framework still has several limitations. First, it relies on pre-treatment scRNA-seq profiles and known drug profiles, which may limit its immediate clinical applicability and prevent direct generalization to previously unseen drugs. Future work will focus on integrating chemical structural embeddings to enable generalization to novel compounds and exploring strategies to reduce dependence on pre-treatment single-cell data. In addition, while our heuristic strategy for combination therapy prediction is effective in practice, it does not explicitly model drug-drug interactions (DDIs). Future work will therefore explore the incorporation of external knowledge bases and DDI-aware modeling strategies to improve the prediction of combination treatment responses. In addition, although the current implementation relies on single-cell data for high-resolution patient stratification, the underlying transfer-learning framework could be extended to bulk transcriptomic data by replacing the single-cell branch during pretraining with large-scale bulk cohorts (e.g., TCGA) and applying the model directly in the bulk setting. Collectively, this study underscores the critical value of high-resolution scRNA-seq data in decoding therapeutic heterogeneity and advancing personalized cancer treatment.

## Supplementary Material

btag269_Supplementary_Data

## Data Availability

The source code for scTAPE is openly available on GitHub (https://github.com/xinliangSun/scTAPE). The single-cell RNA sequencing datasets utilized in this study were retrieved from DRMref (Liu *et al.* 2024). Drug response profiles were derived using the PRISM multiplexed screening assay (Corsello *et al.* 2020) and quantified as the Area Under the dose-response Curve (AUC) across an eight-point concentration range.
