# Supplementary material for: A disentangled transformer-based transfer learning framework to predict patient drug response from tumor single-cell transcriptomics
Source: Bioinformatics. 2026 Jul 7;42(Suppl 1):btag269. doi: 10.1093/bioinformatics/btag269 (PMC13341125; doi:10.1093/bioinformatics/btag269)
Supplement: btag269_Supplementary_Data [file btag269_supplementary_data.pdf]

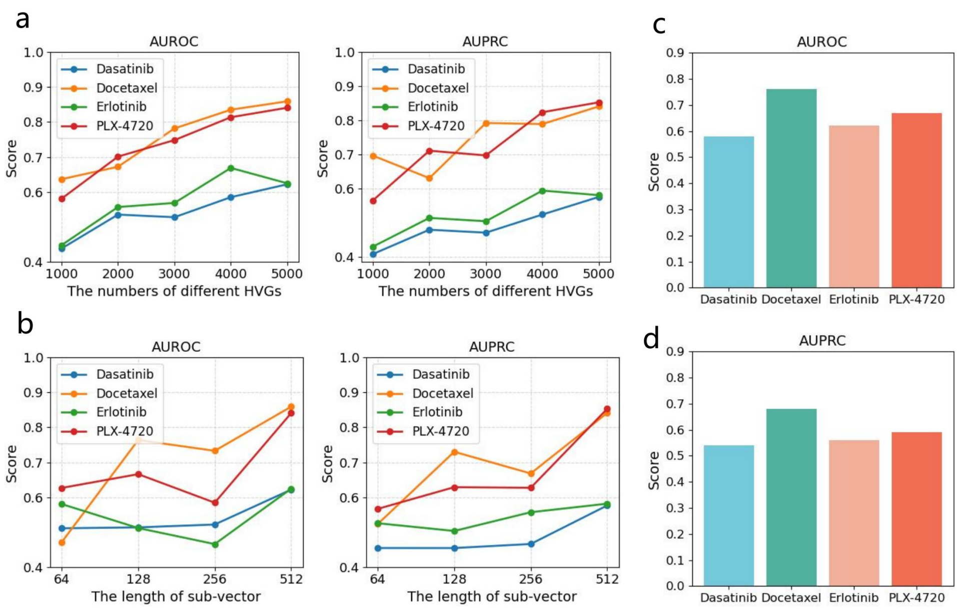

Supplementary Figure S1. (a) AUROC and AUPRC across different numbers of HVGs. (b) AUROC and AUPRC across different sub-vector lengths. (c) AUROC for the scGPT-based model variant. (d) AUPRC for the scGPT-based model variant.

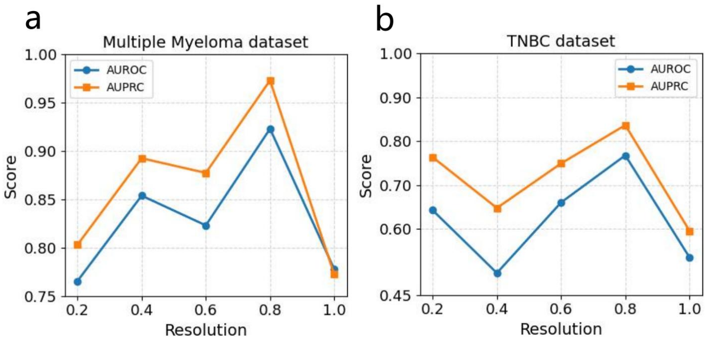

Supplementary Figure S2. (a) AUROC and AUPRC across different resolutions in the multiple myeloma dataset. (b) AUROC and AUPRC across different resolutions in the TNBC dataset.

Supplementary Table S1

Stage	Data used	Size
Pre-training	All CCLE+PRISM bulk cell lines + all SCP single-cell cell lines	1840 bulk cell lines + 194 single-cell cell lines
Fine-tuning	Labeled CCLE+PRISM bulk cell lines	1840 cell lines
Evaluation	Independent external scRNA-seq and clinical datasets	4 cell-line datasets + 2 clinical cohorts

Supplementary Tables 2

	h_dim	z_dim	epoch	lam1	mbS	mbT
Dasatinib	1024	256	100	2	8	8
Docetaxel	512	256	40	3	32	32
Erlotinib	512	256	100	3	32	32
PLX4720	512	256	10	1	8	8

Supplementary Tables 3

	bottleneck	Encoder dims	Predictor dims	dropout	model	lr	Sampling
Docetaxel	512	256,128	256,128	0.1	DAE	0.01	upsampling
Dasatinib	512	512,256	256,128	0.1	DAE	0.01	upsampling
Erlotinib	64	512,256	256,128	0.3	DAE	0.01	upsampling
PLX4720	256	512,256	256,128	0.1	DAE	0.01	upsampling
